# Ligand Local Rigidity as a Quantitative Descriptor of Lanthanide Size Selectivity

**DOI:** 10.1021/acs.inorgchem.6c05141

**Published:** 2026-10-19

**Authors:** Anton S. Pozdeev, Santa Jansone-Popova, De-en Jiang

**Affiliations:** † Department of Chemical and Biomolecular Engineering, 5718 Vanderbilt University, Nashville, Tennessee 37235, United States; ‡ Chemical Sciences Division, 6146 Oak Ridge National Laboratory, Oak Ridge, Tennessee 37831, United States

## Abstract

Separation of the rare-earth elements relies on the small, monotonic contraction in the ionic radius across the trivalent lanthanide series. Ligand preorganization is a powerful strategy for amplifying this size difference, but within the N,O-tetradentate ligand family examined here, it approaches a structural ceiling as the most preorganized scaffolds become fully fused. Here, using density functional theory, we introduce cavity rigidity as a quantitative framework for lanthanide size selectivity and define local rigidity (LR) as the resistance of the ligand cavity to the compression required for metal binding. The LR reflects both the intrinsic stiffness of the ligand framework and the extent to which the cavity must deform upon coordination. Across the N,O-tetradentate ligands examined, the computed La/Lu size selectivity increases linearly with LR. The same relationship is observed for five additional lanthanide pairs with larger ionic radius differences producing steeper slopes. Within this ligand family, the results point to two routes for increasing thermodynamic size selectivity: increasing the framework stiffness or increasing the equilibrium donor separation.

## Graphical abstract

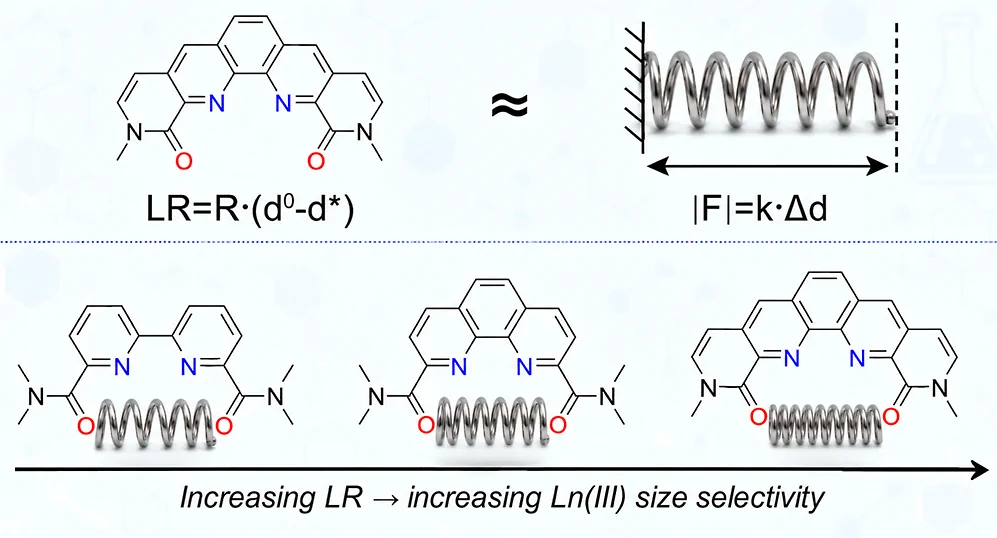

## Introduction

Rare-earth elements—the lanthanides together with scandium and yttrium—are fundamental to technologies central to clean energy, communications, medicine, and national security,^1^ and improving their separation is recognized as a breakthrough that could reshape the chemical industry.^2^ Across the lanthanide series, these ions exhibit remarkably similar coordination chemistry in the +3 oxidation state, while their ionic radii decrease systematically from La to Lu as a result of the lanthanide contraction.^3,4^ Industrial separation still relies predominantly on solvent extraction, which exploits these subtle differences in ionic radius but typically provides only modest selectivity between adjacent lanthanides. As a consequence, it requires long multistage cascades and large volumes of acids and reagents, generating substantial chemical waste.^5^ Addressing these limitations requires extractants that amplify the subtle size differences arising from the lanthanide contraction into enhanced thermodynamic selectivity.^6,7^

Among the strategies developed to enhance lanthanide size discrimination, preorganization has emerged as a powerful design principle, particularly for phenanthroline- and bipyridine-based neutral N,O-donor extractants.^8^ A substantial body of experimental and computational work on these ligand families has shown that lanthanide extraction and selectivity are highly sensitive to conformational constraint, donor-group orientation, chelate geometry, and the extent of structural reorganization accompanying metal binding.^9–16^ Preorganization connects several of these effects by restricting conformational flexibility and biasing the free ligand toward a donor geometry compatible with metal coordination. This reduces the structural reorganization required for binding and can amplify the small differences in binding caused by the lanthanide contraction.^17^ In the broader coordination chemistry of metal-ion recognition, the same principle is expressed as complementarity: discrimination between ions of different size is controlled by the geometry of the donor set—cavity size, chelate ring size, and donor-group orientation—and by the extent to which the framework can distort away from that geometry.^18,19^ Expanded macrocyclic chelators such as macropa provide a direct illustration: rigidification of the macrocyclic backbone enhances selectivity for the larger, light lanthanides, an effect attributed to a rigid framework that cannot distort sufficiently to accommodate smaller ions.^20^ Within the N,O-tetradentate family, progressive rigidification from diamide-bipyridine to diamide-phenanthroline and bis-lactam-phenanthroline scaffolds strongly influences lanthanide binding and separation behavior. Experimental separation factors for DAPhen and BLPhen are also time-dependent, indicating that the observed separation performance reflects not only the underlying thermodynamic discrimination but also kinetic and phase-dependent contributions.^21–24^

Although preorganization has proven to be highly effective, its use as a design principle has two important limitations. First, preorganization remains largely qualitative:^25,26^ although reducing conformational flexibility often improves size discrimination, it does not predict how selectivity will change following structural modification. Moreover, within this N,O-tetradentate ligand family, the most preorganized scaffolds are already fully fused, leaving little scope to eliminate additional conformational degrees of freedom.^23^ Second, even a more quantitative description based on ligand reorganization energy remains incomplete for size selectivity. Reorganization energy measures the energetic cost of deforming the free ligand into its metal-bound geometry for a given ion, whereas size selectivity depends on how this deformation penalty changes between ions of different size.^8,27,28^ We therefore focus on resistance to contraction: ligands that are harder to compress should more strongly disfavor smaller lanthanides. Importantly, this resistance cannot be predicted from the cavity size alone.

Herein, we introduce ligand cavity rigidity as a quantitative framework for the lanthanide size selectivity. We define local rigidity (LR) as the resistance of the coordination cavity to the deformation required for metal binding, combining the mechanical stiffness of the ligand with the extent of the cavity compression. Across the N,O-tetradentate ligands examined, the computed size selectivity for multiple lanthanide pairs correlates strongly with LR, revealing two routes to enhanced discrimination: tuning the mechanical resistance of the framework or the equilibrium geometry of the binding cavity. Within the ligand family examined here, LR thus provides a quantitative mechanical framework for describing size selectivity beyond conventional qualitative measures of preorganization, while its applicability to other donor sets and coordination motifs defines the next level of generalization.

## Results and Discussion

### Structural Limits of Ligand Preorganization

The representative ligand classes shown in Figure 1A can be organized along two dimensions: the conformational stiffness of the binding framework and whether the ligand favors larger or smaller lanthanides. For flexible extractants, the direction of lanthanide preference varies with the ligand class. Acidic organophosphorus reagents generally favor the smaller, heavier lanthanides, consistent with a charge-density-driven trend, whereas malonamides may show no consistent class-wide preference toward either end of the lanthanide series.^29,30^ Octadecyl acyclopa (ODA), despite its flexible and acyclic framework, preferentially extracts the larger, light lanthanides.^31^ The rigid phenanthroline platform also supports different directions of the selectivity. Preorganized phenanthroline diamides favor the lighter lanthanides, whereas NaDOAPA, in which one neutral amide donor is replaced by an anionic carboxylate, shows increasing preference for the heavier lanthanides.^23,32^ These examples show that framework stiffness alone does not determine the direction of lanthanide preference, which also depends on donor identity and geometry.^33,34^ To examine the mechanical contribution to lanthanide discrimination, we focus on the phenanthroline- and bipyridine-based N,O-tetradentate ligand family, where the donor set, coordination mode, and preference for larger lanthanides remain broadly similar. Within this chemically coherent series, increasing structural restriction is accompanied by a marked enhancement of experimental lanthanide discrimination: under identical extraction conditions (1.0 M HNO_3_, 9 h), (SF_La/Lu_) increases from 1.0 for DABPy to 42.5 for DAPhen and 156.6 for BLPhen.^24^

**Figure 1:**
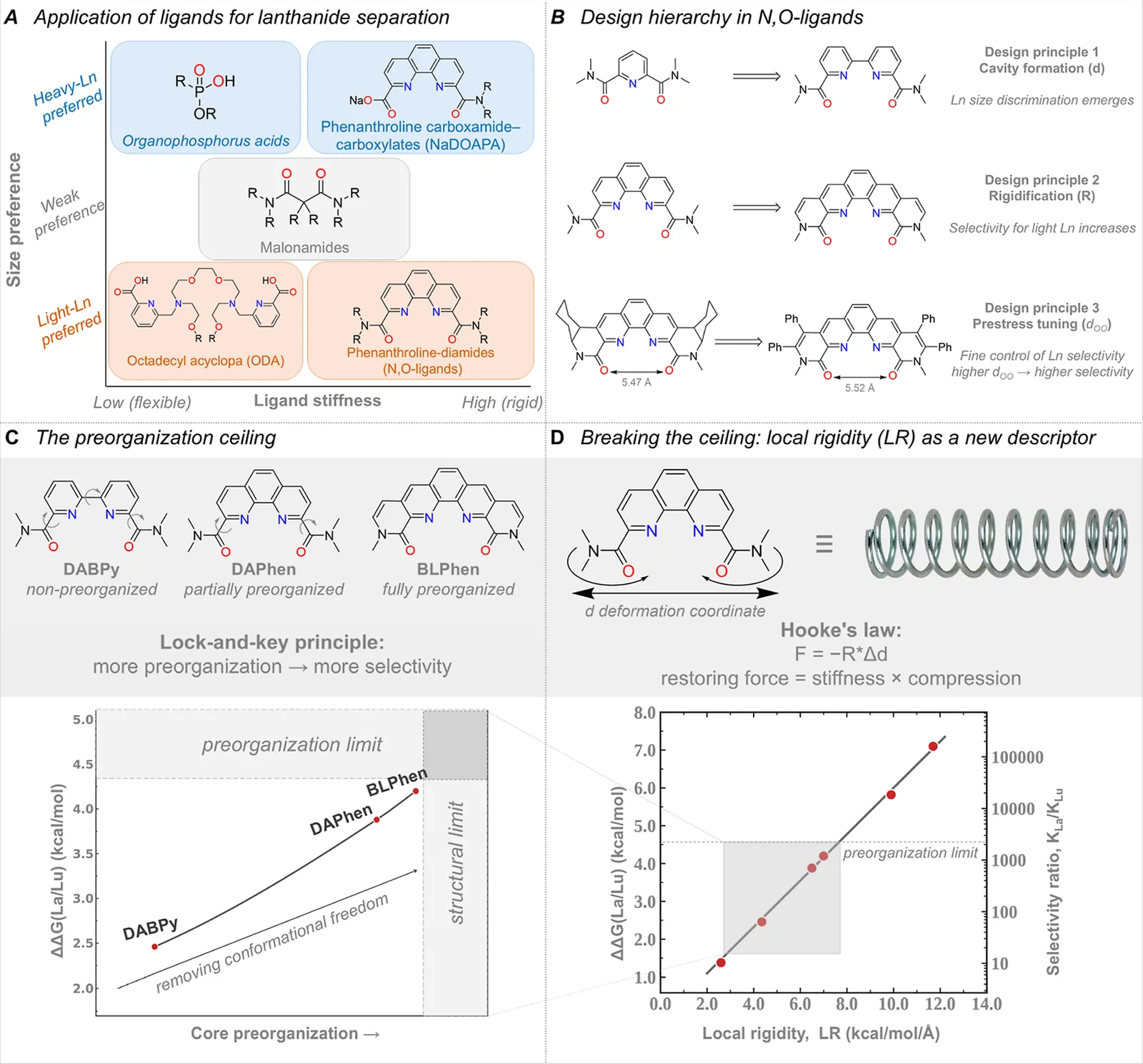
From ligand preorganization to cavity rigidity in lanthanide size selectivity. (A) Representative ligand classes arranged by framework stiffness and lanthanide size preference. (B) Three structural design principles in N,O-tetradentate ligands: formation of the coordination cavity, framework rigidification, and tuning of the equilibrium O···O distance, *d* ^0^. (C) Progressive preorganization increases the computed La/Lu binding free-energy difference, (ΔΔ*G*_(La/Lu)_), but reaches a structural limit at the fully fused BLPhen scaffold. (D) Local rigidity, LR, describes the restoring response of the ligand cavity to compression and provides a quantitative descriptor of ΔΔ*G*_(La/Lu)_ beyond the conventional preorganization limit.

This structural progression can be understood in terms of three design principles (Figure 1B). The first establishes a N_2_O_2_ coordination cavity for size-dependent Ln­(III) binding.^21^ The second increases preorganization by eliminating key rotational degrees of freedom through framework fusion.^22,23^ Even after the framework is fully fused, however, a third tunable feature remains: the equilibrium free-ligand donor atoms (O···O) separation. Variations in this distance have been associated with changes in lanthanide selectivity.^23,24^ Previous studies have discussed such effects in terms of bite size, preorganization, and ligand strain, but these concepts do not directly quantify how strongly the cavity resists contraction. This limitation motivates a new mechanical description of the ligand response to changes in cavity size.

This connection between preorganization, ligand reorganization, and size selectivity is evident within this family: progressive backbone fusion along DABPy (diamide-bipyridine) → DAPhen (diamide-phenanthroline) → BLPhen (bis-lactam-phenanthroline) removes rotatable degrees of freedom while increasing the computed La/Lu binding free-energy difference, ΔΔ*G*_(La/Lu)_, from 2.46 to 3.88 to 4.20 kcal/mol, respectively (Figure 1C). At 298 K, these differential free energies correspond to La/Lu binding-equilibrium constant ratios of approximately 64, 700, and 1200, respectively, illustrating the substantial thermodynamic discrimination represented by differences of only a few kcal/mol. This trend, however, reaches a structural limit once the framework is fully fused, as in BLPhen. Beyond this point, further size discrimination is expected to depend on how strongly the cavity resists contraction at the metal-bound geometry. We quantify this response below using the local restoring force (Figure 1D).

### Defining and Computing Cavity Rigidity

To quantify cavity rigidity, we performed relaxed constrained compression of the free-ligand coordination cavity. Starting from the relaxed free-ligand reference structure, the donor–donor O···O distance, *d*, was decreased stepwise while all remaining degrees of freedom were optimized (Figure 2C), allowing the accompanying response of the N···N separation to evolve freely. This coupled N···N response was monitored for representative bipyridine- and phenanthroline-based ligands and is analyzed in Section S4 of the Supporting Information. The analysis was restricted to compression, reflecting the progressive decrease in the cavity size required across the lanthanide series. Along this compression coordinate, the deformation energy changes smoothly with *d* and is well described within the harmonic region by a quadratic function, *E*(*d*) ≈ *ad* ^2^ + *bd* + *c*.

**Figure 2:**
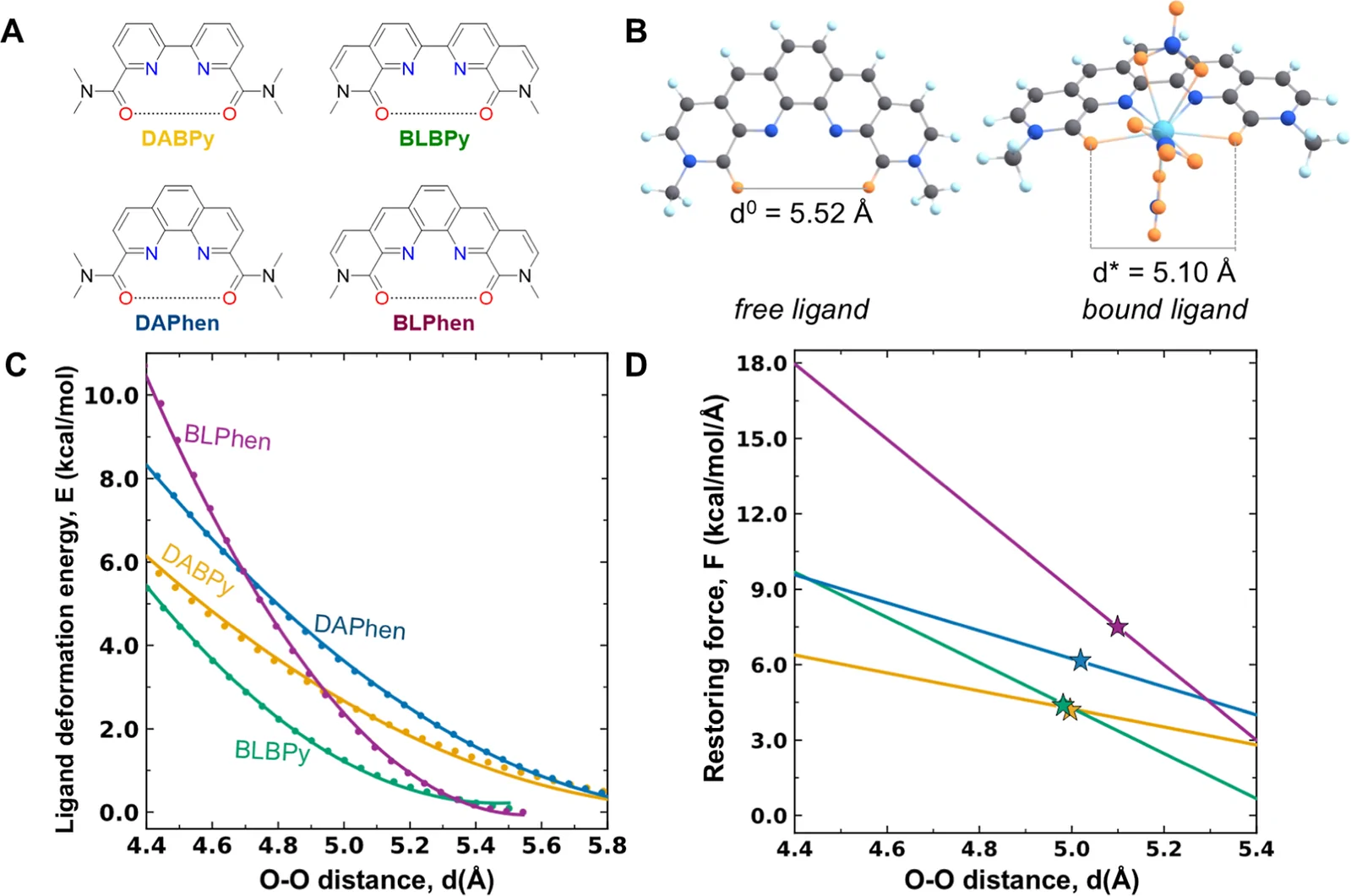
Definition of cavity rigidity descriptors. (A) Representative ligands used to illustrate the rigidity framework and the donor–donor (O–O) distance, *d*, used as the deformation coordinate. (B) Compression of the free-ligand O···O distance from its equilibrium value (*d* ^0^ = 5.52 Å) to the value adopted in the optimized La­(III)-bound geometry (d* = 5.10 Å), illustrated for BLPhen. (C) Deformation-energy profiles, *E*(*d*), obtained by constrained compression of the free ligand. (D) Restoring-force profiles, *F* = −∂*E*/∂*d*, corresponding to panel C; stars mark the values at the La-bound distance, d*, which define the LR.

Two rigidity descriptors are obtained from the deformation profile. Here, *d* ^0^ denotes the equilibrium O···O distance of the free ligand, corresponding to the minimum of the fitted deformation–energy profile, whereas d* denotes the O···O distance adopted by the ligand in the optimized La­(III) complex. For the quadratic deformation profile, the global rigidity, **R**, is defined as its curvature

1 $$R=\frac{\partial^{2}E}{\partial d^{2}}=2a$$

and measures the intrinsic stiffness of the ligand framework along the O···O compression coordinate. The equilibrium free-ligand distance is obtained from the minimum of the quadratic profile

2 $$d^{0}=-\frac{b}{2a}$$

The local rigidity, **LR**, is defined as the restoring force opposing compression evaluated at the La-bound distance, (*d* = d*)

3 $$LR=-{\frac{\partial E}{\partial d}|}_{d=d^{*}}=R\left(d^{0}-d^{*}\right)$$

The corresponding restoring-force profiles are shown in Figure 2D. Within the harmonic region, these profiles are linear, with the magnitude of the slope reflecting the global rigidity *R*; the star symbols mark the restoring force at the La-bound distance d*, which defines LR for each ligand. Thus, LR depends jointly on the intrinsic stiffness of the ligand framework and the displacement of the cavity from its equilibrium geometry upon binding. Consequently, the same LR can arise from a stiffer framework, a larger cavity compression, or a combination of the two.

The computed global rigidities increase in the order DABPy (3.35) < DAPhen (5.61) < BLBPy (9.02) < BLPhen (16.83 kcal/mol/Å^2^). Fusion of the bipyridine core to form phenanthroline produces a relatively modest increase in intrinsic cavity stiffness, whereas cyclization of the diamide groups into fused bis-lactams produces a substantially larger increase. BLPhen, in which both regions are fused, is therefore the stiffest member of this series.

The ordering changes when the restoring force is evaluated at the La-bound cavity size: DABPy (4.17) < BLBPy (4.44) < DAPhen (6.13) < BLPhen (7.05 kcal/mol/Å). The origin of this change is illustrated by the BLBPy/DAPhen comparison: although BLBPy is intrinsically stiffer than DAPhen (*R* = 9.02 versus 5.61 kcal/mol/Å^2^), its equilibrium cavity lies closer to the La-bound geometry and therefore develops a smaller restoring force. DAPhen has a softer framework but undergoes greater cavity displacement, resulting in the higher LR. This inversion motivates testing LR as the mechanical descriptor of lanthanide size selectivity.

### Local Rigidity as a Quantitative Descriptor of Size Selectivity

Across the 13 ligands examined, the computed differential binding free energy, ΔΔ*G*_(La/Lu)_, varies linearly with LR, ΔΔ*G*_(La/Lu)_ = 0.591·LR + 0.162 (*R* ^2^ = 0.992; Figure 3A). The ligand set was chosen to sample both structural and donor-group diversity within the common N_2_O_2_ coordination motif. In addition to amide- and lactam-based ligands, it includes previously reported N-oxide-based N,O-donor structures,^35^ allowing the LR–selectivity relationship to be tested across chemically distinct O-donor environments. Highly fused polycyclic scaffolds were additionally introduced as hypothetical models to extend the LR range into the extreme-rigidity regime. The persistence of the same linear relationship despite variations in O-donor chemistry and scaffold architecture within this N_2_O_2_ ligand series indicates that LR captures a common mechanical contribution to size selectivity.

**Figure 3:**
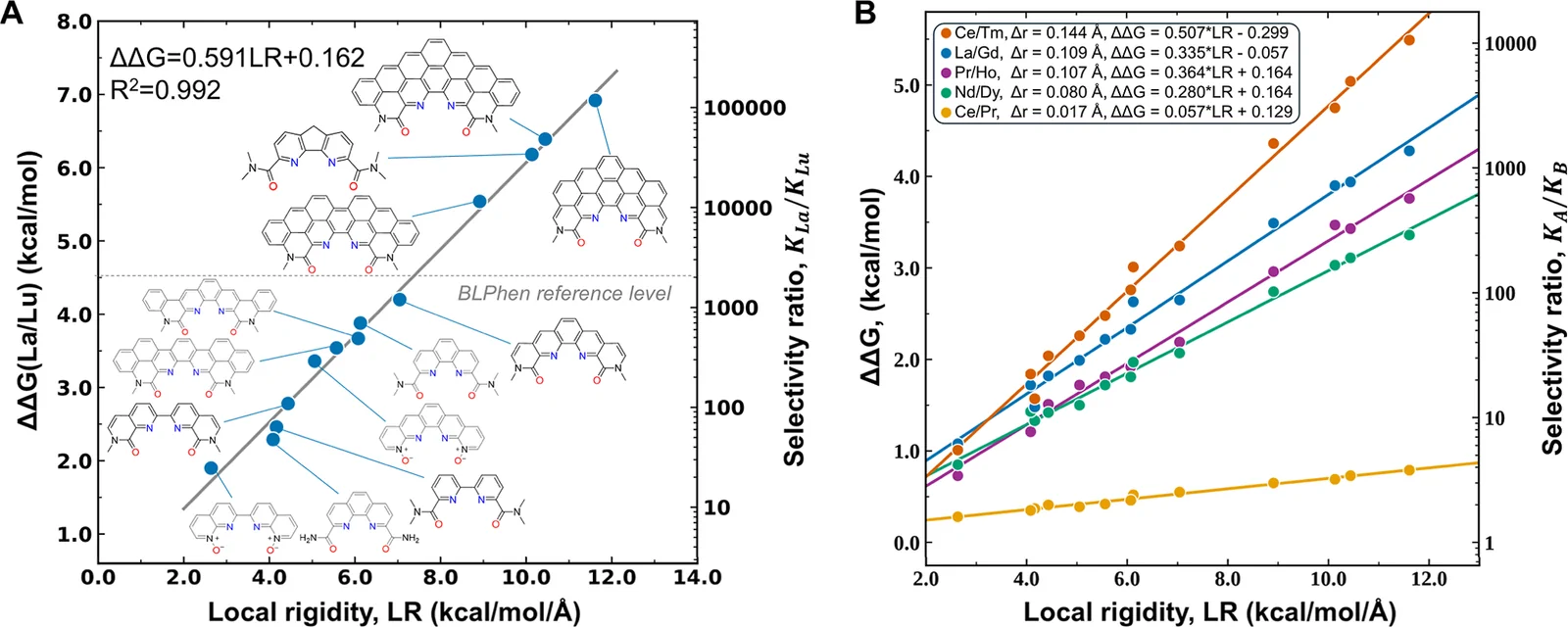
LR–selectivity relationships across lanthanide pairs. (A) Linear relationship between computed ΔΔ*G*_(La/Lu)_ and LR across the 13 ligands (ΔΔ*G* = 0.591·LR + 0.162, *R* ^2^ = 0.992). (B) Corresponding relationships between ΔΔ*G*(A/B) and LR for five additional lanthanide pairs: Ce/Tm, La/Gd, Pr/Ho, Nd/Dy, and Ce/Pr. Ionic-radius differences, Δ*r*, were calculated using Shannon radii for nine-coordinate Ln­(III) ions.

The statistical robustness of the LR–selectivity relationship was assessed using leave-one-out cross-validation and influence analysis (Section S5, Supporting Information). The relationship remained essentially unchanged upon exclusion of individual ligands (*Q*_LOO_^2^ = 0.989; maximum change in slope, 1.7%). In addition, a regression constructed using only the eight ligands not introduced specifically to extend the LR range predicted the five withheld BLPhen-B_n_ models with an RMSE of 0.09 kcal/mol. The LR–selectivity relationship is likewise robust to the electronic-structure treatment, remaining strongly linear across five additional DFT protocols spanning GGA, *meta*-GGA, hybrid-GGA, and hybrid-*meta*-GGA functionals (*R* ^2^ = 0.988 – 0.997; *Q*_LOO_^2^ = 0.971 – 0.995; Section S6, Supporting Information). Importantly, this strong correlation is not reproduced by global rigidity alone. Global rigidity, *R*, shows only a weak correlation with ΔΔ*G*_(La/Lu)_ (*R* ^2^ = 0.336, Figure S4), indicating that intrinsic framework stiffness alone does not account for the observed variation in size selectivity. The strong correlation with LR therefore reflects the combined influence of framework stiffness and the cavity deformation required to reach the metal-bound geometry.

This relationship places conventional preorganization within a broader mechanical description of size selectivity. The progression DABPy → DAPhen → BLPhen traces a trajectory along the LR–selectivity relationship in which progressive conformational restriction increases both LR and ΔΔ*G*_(La/Lu)_. In this view, preorganization enhances size discrimination not only by restricting the accessible conformational space but also by increasing the restoring force generated as the ligand cavity is compressed toward its metal-bound geometry. The DAPhen → BLPhen transformation illustrates this effect directly: cyclization of the diamide groups into the fused bis-lactam framework increases the energetic penalty for O···O compression and therefore the intrinsic cavity stiffness, *R*. More generally, LR can be increased through either a greater mechanical stiffness of the framework or a larger displacement between the equilibrium and metal-bound cavity geometries, *d* ^0^ – d*.

Complete conformational preorganization does not, however, exhaust the structural control of cavity mechanics. Experimental studies of closely related BLPhen ligands illustrate this point: under identical extraction conditions (0.9 M HNO_3_, 1,2-dichloroethane, 25 h), two preorganized BLPhen derivatives differing in lactam-ring saturation exhibit SF_(La/Lu)_ values of 17,326 and 495. The distinct selectivity profiles have been associated with differences in ligand preorganization and rigidity, together with changes in donor strength and complex speciation.^23^ Within the fully fused BLPhen-derived structures examined here, additional annulation provides a direct means of modifying the mechanical response of the coordination cavity. The added cyclic constraints couple O···O compression to concerted bond-angle and bending distortions across the fused scaffold, with the resulting deformation penalty depending on the position and connectivity of the additional rings. These changes are reflected in substantial variations in *R*, while differences in the equilibrium donor geometry further modulate the displacement *d* ^0^ – d*. Fully preorganized ligands can therefore span a broad range of LR values and remain distributed along the same LR–ΔΔ*G*_(La/Lu)_ relationship as the less constrained members of the series.

To assess whether the LR–selectivity relationship extends across lanthanide pairs within the same ligand space, we evaluated ΔΔ*G* for five additional lanthanide pairs: Ce/Tm, La/Gd, Pr/Ho, Nd/Dy, and Ce/Pr. The pairs were selected to span a wide range of size mismatches, from the adjacent Ce/Pr pair to widely separated ions, while also including La/Gd and Pr/Ho, which have nearly identical Δ*r* values despite occupying different regions of the lanthanide series. For every pair, ΔΔ*G* varies linearly with the same ligand-specific LR values (*R* ^2^ = 0.983–0.994, Figure 3B). Moreover, the fitted slope increases approximately with the corresponding ionic-radius difference, Δ*r*, calculated from Shannon radii for coordination number nine.^3^

For each lanthanide pair, the dependence of size selectivity on LR can be written as ΔΔ*G*(A/B) = *m*_A/B_LR + *b*_A/B_. The fitted slope, *m*_A/B_, increases approximately in proportion to the ionic-radius difference, Δ*r*, while the intercept remains pair-dependent, such that the LR-dependent contribution to selectivity can be expressed to first order as *k*Δ*r*·LR (*k* = 3.257, Figure S3). This result suggests an approximate separation of ligand and metal-pair effects: LR describes the mechanical resistance of the ligand cavity to contraction, whereas Δ*r* sets the magnitude of the size mismatch between the two ions. For the N,O-tetradentate series examined here, LR thus provides a common mechanical descriptor of thermodynamic size selectivity across multiple lanthanide pairs.

Within the harmonic regime, the cavity deformation profile admits a simple spring analogy that motivated the present rigidity framework and provides an intuitive interpretation of its design variables (Figure 4A). Progressively smaller lanthanide ions require greater compression from the equilibrium cavity size, *d* ^0^, corresponding to a larger restoring force and a deformation penalty. For an ideal spring, the restoring force is given by |*F*| = *kx*, where *k* is the spring constant and *x* is the displacement from the equilibrium. Within the harmonic region used to define LR, the relaxed cavity response can be expressed in the same linear form as an ideal spring, with *R* acting as the effective spring constant and *d* ^0^ – d* representing the displacement from equilibrium. Stiffening the framework increases the spring constant, *R* (Figure 4B), whereas expanding the equilibrium donor separation increases the displacement, *d* ^0^ – d*, required to reach the metal-bound geometry (Figure 4C). Conventional framework rigidification primarily exploits the first route and approaches a structural limit once conformational locking is exhausted; the second route remains available through the tuning of the equilibrium cavity geometry.

**Figure 4:**
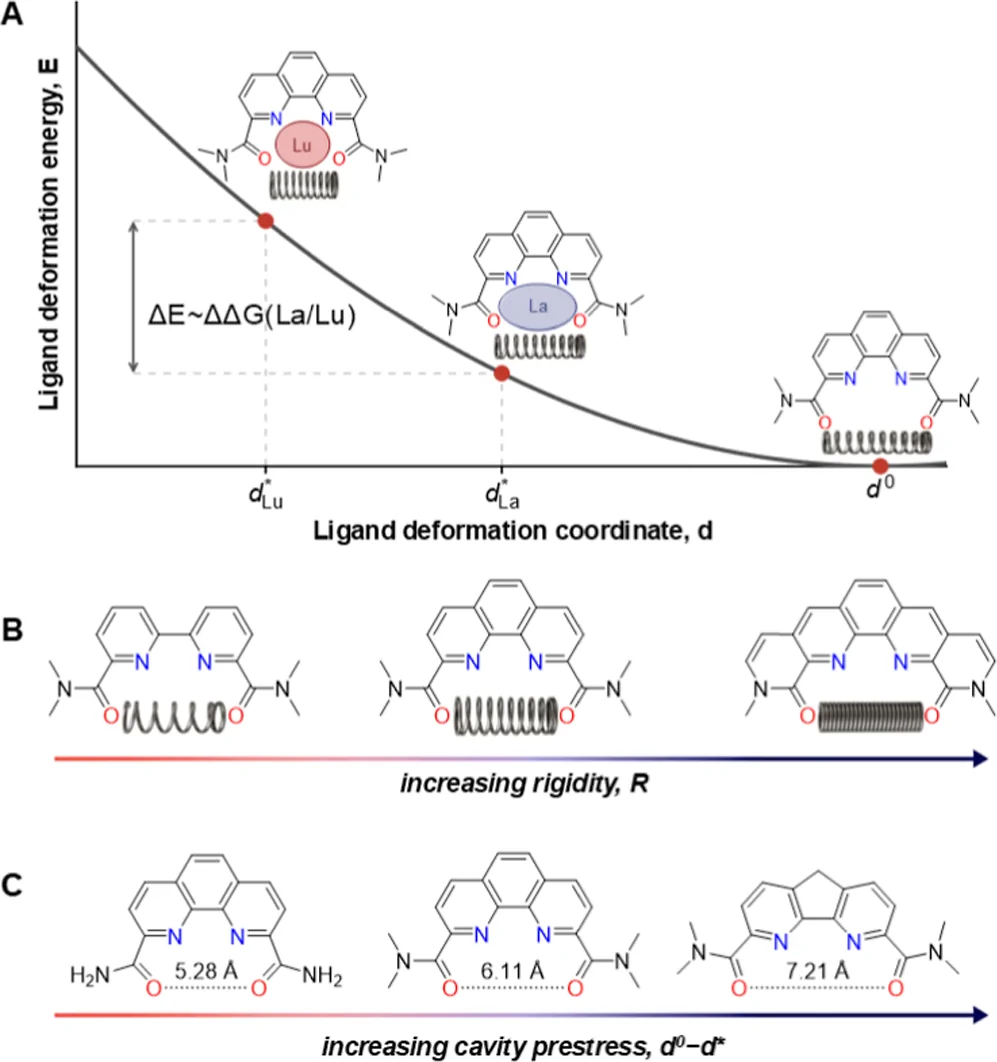
Mechanical basis of lanthanide size selectivity and two routes to increasing LR. (A) Ligand deformation associated with compression from the equilibrium cavity size, *d* ^0^, to the La- and Lu-bound geometries, *d*_La_^*^ and *d*_Lu_^*^. LR can be increased either by (B) increasing the intrinsic framework rigidity, *R*, or by (C) increasing cavity prestress, *d* ^0^ – d*, through expansion of the equilibrium donor geometry.

### Scope and Limitations

The present analysis establishes the LR–selectivity relationship within a chemically related series of N,O-tetradentate ligands sharing a common N_2_O_2_ donor set and coordination motif. Within this scope, LR provides a quantitative description of the mechanical contribution to thermodynamic size selectivity arising from the response of the coordination cavity to metal binding. Separation performance, however, is governed by additional factors that are not captured by the equilibrium deformation–energy profile, including absolute binding affinity, solubility and phase partitioning, complexation and stripping kinetics,^36–38^ stability under strongly acidic or radiolytic conditions, and susceptibility to aggregation or third-phase formation.^39–41^ These factors must therefore be evaluated independently and may introduce practical trade-offs between rigidity, binding affinity, kinetic accessibility, and phase behavior. Extending the LR framework to other donor sets and coordination modes will determine how broadly this mechanical description of ligand-controlled size selectivity can be generalized.

## Conclusion

This work introduces cavity rigidity as a quantitative mechanical framework for thermodynamic lanthanide size selectivity. By expressing LR as LR = *R*(*d* ^0^ – d*), the framework links intrinsic scaffold stiffness with the cavity deformation required for binding. Across 13 N,O-tetradentate ligands and six lanthanide pairs, computed selectivity follows a common LR-based relationship, with pair-specific slopes increasing approximately with ionic-radius difference. Within the ligand space examined here, this relationship identifies two complementary routes to enhanced size discrimination—stiffening the ligand framework and tuning the equilibrium donor geometry—and extends ligand design beyond the structural limits of conventional preorganization. More broadly, it reframes size recognition as a problem of binding-cavity mechanics as well as geometry, providing a physically grounded connection between molecular structure and thermodynamic selectivity. The present results establish this framework within a structurally and chemically related N,O-tetradentate ligand family; its transferability to other donor sets and coordination motifs remains to be determined. Translation of this principle to practical liquid–liquid separations will additionally require consideration of binding affinity, extraction kinetics, phase behavior, and chemical stability.

## Computational Methods

All DFT calculations were performed with ORCA 6.1.1.^42,43^ Geometry optimizations employed the TPSS functional^44^ with the D4 dispersion correction^45^ and the def2-TZVP basis set^46^ for all nonlanthanide atoms. Lanthanide ions were described using Stuttgart–Dresden quasirelativistic 4f-in-core pseudopotentials^47,48^ and the corresponding segmented valence basis sets.^49^ Aqueous solvation was represented using the SMD continuum model,^50^ and Gibbs free energies were evaluated at 298.15 K using ORCA’s quasi-rigid-rotor harmonic-oscillator treatment of low-frequency vibrational modes.^51^

All Ln­(III) complexes were modeled as ten-coordinate Ln­(L)­(NO_3_)_3_ species, with the tetradentate ligand and three bidentate nitrate anions completing the first coordination sphere. Free-ligand conformational space was explored using CREST^52,53^ at the GFN2-xTB/ALPB­(water) level,^54,55^ followed by DFT reoptimization. The lowest-energy conformer was used for binding thermodynamics, whereas cavity deformation calculations employed the relaxed free ligand extracted from the corresponding La­(L)­(NO_3_)_3_ complex.

Binding free energies, Δ*G*_b_, were evaluated for the aqueous ligand–exchange reaction

$$L_{\left(aq\right)}+Ln{\left({NO}_{3}\right)}_{3}{(H_{2}{O)}_{3\left(aq\right)}\to Ln\left(L\right)\left({NO}_{3}\right)}_{3\left(aq\right)}+{3H}_{2}O_{\left(aq\right)}$$

Size selectivity was defined as ΔΔ*G*(A/B) = Δ*G*_b_(B) – Δ*G*_b_(A), where A is the larger lanthanide ion, so that positive values indicate preferential binding of the larger ion.

Cavity-compression scans were performed by decreasing the O···O distance in steps of 0.05 Å while relaxing all remaining geometric degrees of freedom. The resulting deformation–energy profiles were fitted to a quadratic function within a contiguous harmonic window containing the La-bound distance, d*, with its lower boundary determined by the onset of systematic deviations from quadratic behavior. The rigidity descriptors *R*, *d* ^0^, and LR (eqs 1–3) were obtained from these fits. Further details of electronic-structure calculations (Section S1.1), conformational sampling and ligand reference states (Section S1.2), complex models and binding thermodynamics (Section S1.3), cavity-compression scans and rigidity descriptors (Section S1.4), and harmonic fitting-window selection (Section S1.5) are provided in the Supporting Information.

## Supplementary Material





## Notes

Computational details; ligand reference states and binding thermodynamics; cavity-compression scans, quadratic-fit parameters, and harmonic fitting-window selection with residual analysis; structures of the thirteen tetradentate ligands; tabulated rigidity descriptors and differential binding free energies; dependence of the LR–selectivity slope on ionic-radius difference; N···N response during cavity compression; leave-one-out cross-validation, influence, and holdout analyses; robustness of the LR–selectivity relationship across DFT levels of theory; and global rigidity–selectivity relationship (PDF)
Cartesian coordinates of optimized structures (XYZ)
