# Supplementary material for: Ligand Local Rigidity as a Quantitative Descriptor of Lanthanide Size Selectivity
Source: Inorg Chem. 2026 Oct 19;65(43):25616–23. doi: 10.1021/acs.inorgchem.6c05141 (PMC13733266; doi:10.1021/acs.inorgchem.6c05141)
Supplement: Supplementary file 1 [file ic6c05141_si_001.pdf]

Supporting Information

Ligand Local Rigidity as a Quantitative Descriptor of Lanthanide Size Selectivity

Anton S. Pozdeev,[a,*] *Santa Jansone-Popova,*[b] *and De-en Jiang*[a,*]

[a] Department of Chemical and Biomolecular Engineering, Vanderbilt University, Nashville, Tennessee 37235, United States

[b] Chemical Sciences Division, Oak Ridge National Laboratory, Oak Ridge, Tennessee 37831, United States

***Corresponding Authors:** *anton.s.pozdeev@vanderbilt.edu; de-en.jiang@vanderbilt.edu*

Contents

S1. Computational Details S2
S1.1. Electronic-Structure Calculations S2
S1.2. Conformational Sampling and Ligand Reference States S2
S1.3. Complex Model and Binding Thermodynamics S2
S1.4. Cavity-Compression Scans and Rigidity Descriptors S3
S1.5. Representative Determination of the Harmonic Fitting Window S4
S2. Ligand Structures S6
S3. Rigidity Descriptors and Lanthanide Size Selectivity S7
S4. N···N Response during Cavity Compression S10
S5. Leave-One-Out Cross-Validation of the LR–Selectivity Relationship S10
S6. Robustness of the LR–Selectivity Relationship across DFT Levels of Theory S11
S7. Global Rigidity–Selectivity Relationship S13
S8. Cartesian Coordinates S13
Supporting References S14

S1. Computational Details

S1.1. Electronic-Structure Calculations

All DFT calculations were performed with ORCA 6.1.1. [1,2] Geometry optimizations employed the TPSS functional with the D4 dispersion correction [3,4] and the def2-TZVP basis set [5] for all non-lanthanide atoms. The resolution-of-identity approximation was used together with the def2/J auxiliary basis sets. [6] Lanthanide ions were described using Stuttgart–Dresden quasirelativistic 4f-in-core pseudopotentials [7,8] and the corresponding segmented valence basis sets, [9] with auxiliary basis sets generated using AutoAux. [10] All DFT calculations included aqueous solvation through the SMD continuum model. [11] Gibbs free energies were evaluated at 298.15 K using ORCA's quasi-rigid-rotor harmonic-oscillator treatment of low-frequency vibrational modes. [12]

S1.2. Conformational Sampling and Ligand Reference States

The conformational space of each free ligand was explored using CREST [13,14] at the GFN2-xTB level [15] with the ALPB implicit solvation model parameterized for water. [16] The lowest-energy conformer identified by CREST was subsequently reoptimized at the DFT level described above. Given the sensitivity of computed lanthanide selectivity to the choice of free-ligand conformational reference state, [17] two free-ligand structures were defined for distinct purposes. The resulting DFT-optimized global minimum was used as the free-ligand reference, L, in the binding reaction defined below. In contrast, the cavity-deformation analysis employed the local minimum continuously connected to the coordinated geometry. This structure was generated by extracting the ligand from the optimized $La(L)(NO_3)_3$ complex and relaxing it without constraints. All deformation-derived quantities, including d^0, R, and LR, refer to this complex-derived local minimum. Using the global free-ligand minimum for the deformation scan would introduce conformational-reorganization contributions unrelated to compression of the coordination cavity.

S1.3. Complex Model and Binding Thermodynamics

All Ln(III) complexes were modeled as ten-coordinate $Ln(L)(NO_3)_3$ species, in which the tetradentate ligand and three bidentate nitrate anions complete the first coordination sphere. This model is consistent with experimentally characterized coordination motifs for tetradentate phenanthroline diamide complexes [18,19] and with established computational protocols for evaluating relative binding trends across the lanthanide series. [20,21]

Binding free energies were calculated for the aqueous ligand-exchange reaction

$$L_{(aq)} + \mathrm{Ln(NO_3)_3(H_2O)_3}_{(aq)} \rightarrow \mathrm{Ln(L)(NO_3)_3}_{(aq)} + 3\mathrm{H_2O}_{(aq)} \qquad (S1)$$

according to

$$\Delta G_\mathrm{b} = G[\mathrm{Ln(L)(NO_3)_3}] + 3G[\mathrm{H_2O}] - G[\mathrm{L}] - G[\mathrm{Ln(NO_3)_3(H_2O)_3}] \qquad (S2)$$

Thermodynamic size selectivity between lanthanides A and B, where A is the larger ion, was defined as

$$\Delta\Delta G(A/B) = \Delta G_{\text{b}}(B) - \Delta G_{\text{b}}(A) \qquad \text{(S3)}$$

Accordingly, a positive ΔΔG(A/B) indicates preferential binding of the larger lanthanide ion. The same complex stoichiometry and coordination motif were retained throughout the lanthanide series so that the calculated differences primarily reflect the response of the ligand framework to lanthanide contraction rather than changes in complex composition. Ionic-radius differences, Δr, were calculated using Shannon effective radii for nine-coordinate Ln(III) ions. [22]

S1.4. Cavity-Compression Scans and Rigidity Descriptors

Cavity-deformation scans were initiated from the relaxed complex-derived free-ligand conformer described above. The donor–donor O···O distance, d, was decreased in increments of 0.05 Å, while all remaining geometric degrees of freedom were relaxed at each constrained value. Accordingly, $E(d)$ represents a relaxed one-dimensional projection of the multidimensional deformation surface, in which accompanying changes in donor orientation, backbone torsion, out-of-plane distortion, and other internal coordinates are incorporated through geometry relaxation. Only compression was sampled because contraction of the coordination cavity is the deformation relevant to binding progressively smaller lanthanide ions. The total scanned interval was approximately 2–4 Å, with longer intervals used for the more flexible ligands. The deformation energy, $E(d)$, was referenced to the unconstrained complex-derived local minimum. Because the deformation profiles departed from quadratic behavior under strong compression, each profile was fitted to

$$\mathrm{E(d) = ad^2 + bd + c} \qquad \text{(S4)}$$

only within its harmonic region. The fitting interval was defined as the contiguous set of scan points that included the complexed distance, d^*, and extended to the onset of nonlinearity in the corresponding restoring-force profile. This boundary generally occurred at deformation energies approximately 15–20 kcal/mol above the minimum. All quadratic fits had coefficients of determination of at least 0.998.

The global rigidity, R, was defined as the curvature of the fitted deformation profile:

$$R = \frac{\partial^2 E}{\partial d^2} = 2a \qquad \text{(S5)}$$

The equilibrium cavity size, d^0, was obtained from the vertex of the fitted parabola:

$$d^0 = -\frac{b}{2a} \qquad \text{(S6)}$$

Because $d^0$is derived from the harmonic fit, it does not necessarily coincide exactly with the O···O distance in the unconstrained optimized structure. The local rigidity, LR, was defined as the restoring force evaluated at the O···O distance adopted in the optimized La complex:

$$LR = -\left.\frac{\partial E}{\partial d}\right|_{d=d^*} = R(d^0 - d^*), \qquad \text{(S7)}$$

where $d^*$is the O···O distance in the optimized La(L)(NO_3)$_3$ complex. Thus, R measures the intrinsic stiffness of the free-ligand framework along the cavity-compression coordinate, whereas LR incorporates both this intrinsic stiffness and the displacement of the ligand from its equilibrium cavity size upon complexation.

S1.5. Representative Determination of the Harmonic Fitting Window

Figure S1 illustrates the selection of the harmonic region of the cavity-compression profile using NOBLPhen (Figure S2) as a representative example. The calculated deformation energies were fitted to the quadratic function defined in eq S4 over a contiguous interval containing the metal-bound distance, d^*. The interval was extended toward shorter O···O distances until systematic deviations from quadratic behavior appeared. For NOBLPhen, scan points with $d_{\mathrm{O-O}} \geq 3.889$Å were included in the fit, whereas points at shorter distances were excluded.

Within the selected window, the residuals remained close to zero and within ±0.5 kcal/mol. Under stronger compression, the residuals became systematically negative and the deformation-energy profile became approximately linear, indicating a departure from the harmonic deformation regime. The fitted coefficients were used to calculate R, d^0, and LR according to eqs S5–S7. The same procedure was applied independently to each ligand, with the lower boundary of the fitting window defined by the onset of systematic nonlinearity in the residual profile.

Across all thirteen ligands, application of the same fitting criterion yielded harmonic windows with maximum absolute residuals ranging from 0.049 to 0.367 kcal/mol (Table S1), consistent with the behavior illustrated above for NOBLPhen. In every case, the La-bound distance, d^*, falls within the selected fitting interval, and LR is therefore evaluated within the region described by the quadratic deformation model. Systematic deviations at stronger compression were used to define the lower boundary of the harmonic fitting window.

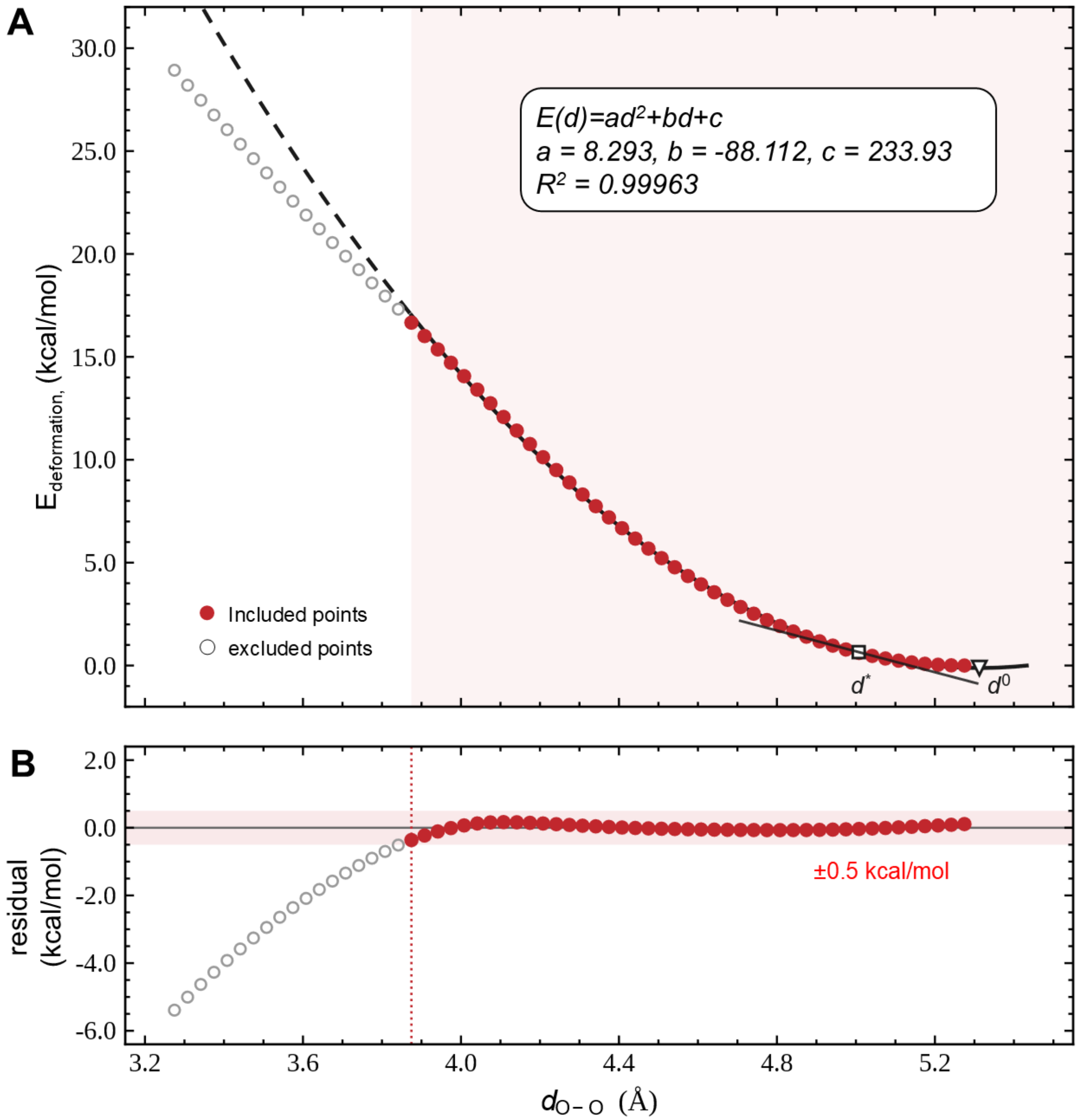

Figure S1. Representative harmonic-window selection for NOBLPhen. (A) Cavity-deformation profile and quadratic fit; filled and open circles denote included and excluded scan points, respectively. d^0 and d^* are the fitted equilibrium and La-bound O···O distances. (B) Corresponding fit residuals.

S2. Ligand Structures

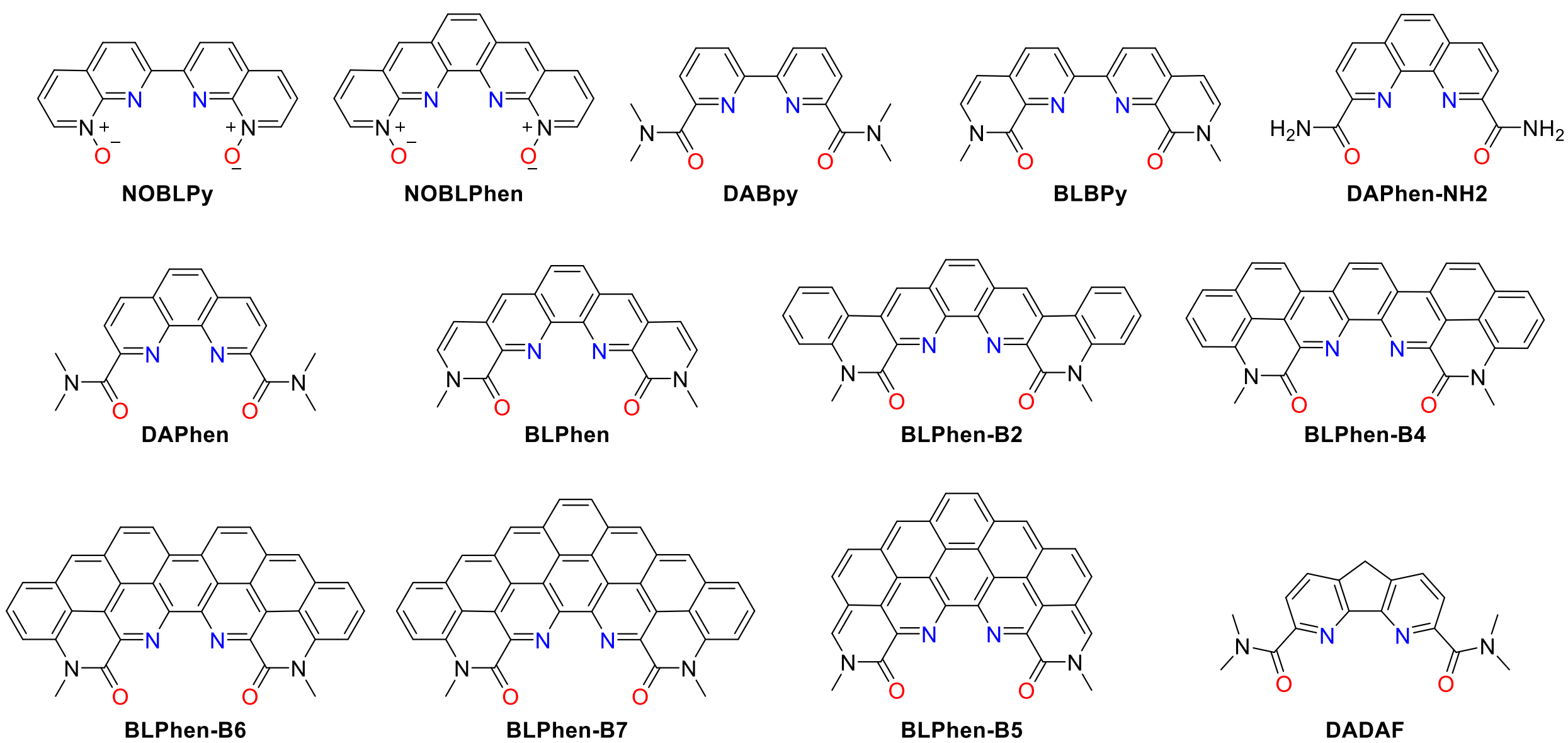

Figure S2. Structures of the thirteen tetradentate ligands considered in this work. Abbreviations follow the scheme [donor][backbone]-Bn: DA = diamide, BL = bis-lactam, and NO = N-oxide; BPy = 2,2′-bipyridine, Phen = 1,10-phenanthroline, and DAF = 4,5-diazafluorene. NH_2 denotes primary amide substituents, and Bn denotes the number of additional fused six-membered rings in the BLPhen-derived backbone.

S3. Rigidity Descriptors and Lanthanide Size Selectivity

Table S1. Quadratic-fit parameters for the free-ligand cavity-compression profiles and the resulting rigidity descriptors.

ligand	a (kcal/mol/Å²)	b (kcal/mol/Å)	c (kcal/mol)	R^2	Fitting range, Å	Max |residual|, kcal/mol	R (kcal/mol/Å²)	d^0 (Å)	d* (Å)	LR (kcal/mol/Å)
**DABPy**	1.6739	-20.884	65.26	0.9998	6.487 - 4.537	0.124	3.35	6.238	4.992	4.17
**BLBPy**	4.5080	-49.353	135.30	0.9999	5.501 - 3.701	0.212	9.02	5.474	4.982	4.44
**DAPhen**	2.8071	-34.305	104.95	0.9999	6.133 - 3.333	0.151	5.61	6.110	5.019	6.13
**BLPhen**	8.4136	-92.883	256.38	0.9998	5.544 - 4.344	0.049	16.83	5.520	5.101	7.05
**NOBLPy**	4.0474	-42.470	111.50	0.9997	5.275 – 4.125	0.136	8.09	5.247	4.920	2.64
**NOBLPhen**	8.2928	-88.112	233.93	0.9996	5.389 – 3.889	0.367	16.59	5.313	5.007	5.06
**DAPhen-NH_2**	6.5806	-69.499	183.58	0.9999	5.294 - 3.894	0.077	13.16	5.281	4.970	4.09
**BLPhen-B2**	8.0330	-87.235	236.63	0.9998	5.483 - 4.083	0.115	16.07	5.430	5.052	6.08
**BLPhen-B4**	8.4098	-90.280	241.99	0.9989	5.385 - 3.985	0.225	16.82	5.368	5.037	5.57
**BLPhen-B6**	10.7296	-119.488	332.25	0.9989	5.568 - 4.368	0.349	21.46	5.568	5.152	8.92
**BLPhen-B7**	12.0010	-135.648	382.94	0.9989	5.663 - 4.563	0.352	24.00	5.652	5.216	10.45
**BLPhen-B5**	13.1694	-150.337	429.01	0.9998	5.723 - 4.773	0.164	26.34	5.708	5.266	11.62
**DADAF**	2.4842	-35.836	129.24	0.9998	7.296 - 4.996	0.124	4.97	7.213	5.171	10.14

Deformation profiles were fitted to $E(d) = ad^2 + bd + c$ within the harmonic window of each relaxed compression scan; R^2 is the coefficient of determination of that fit. $R = 2a$ is the global rigidity, $d^0 = -b/2a$ is the O–O distance at the vertex of the fitted parabola, d* is the O–O distance in the optimized $La(L)(NO_3)_3$ complex, and $LR = R(d^0 - d^*)$ is the local rigidity.

Table S2. Computed differential binding free energies, $\Delta\Delta G(A/B)$, for the thirteen ligands and six lanthanide pairs.

Ln pair	DABPy	BLBPy	DAPhen	BLPhen	NOBLPhen	NOBLPy	DAPhen-NH_2	BLPhen-B2	BLPhen-B4	BLPhen-B6	BLPhen-B7	BLPhen-B5	DADAF	Δr
**La/Lu**	2.46	2.78	3.88	4.20	3.36	1.90	2.29	3.67	3.54	5.54	6.39	6.92	6.18	0.184
**Ce/Tm**	1.57	2.04	3.01	3.24	2.26	1.01	1.84	2.76	2.48	4.36	5.04	5.49	4.75	0.144
**La/Gd**	1.52	1.51	1.96	2.19	1.72	0.73	1.21	1.92	1.81	2.96	3.43	3.76	3.47	0.109
**Pr/Ho**	1.48	1.82	2.63	2.65	1.99	1.08	1.72	2.33	2.22	3.49	3.94	4.28	3.90	0.107
**Nd/Dy**	1.33	1.42	1.97	2.07	1.50	0.85	1.43	1.81	1.72	2.74	3.11	3.36	3.03	0.080
**Ce/Pr**	0.37	0.41	0.52	0.55	0.39	0.28	0.35	0.46	0.42	0.65	0.73	0.79	0.69	0.017

$\Delta\Delta G$ values are reported in kcal/mol and Δr values in Å. Differential binding free energies were calculated as $\Delta\Delta G(A/B) = \Delta G_{\mathrm{b}}(B) - \Delta G_{\mathrm{b}}(A)$, where A is the larger lanthanide ion. Positive values therefore indicate preferential binding of the larger ion.

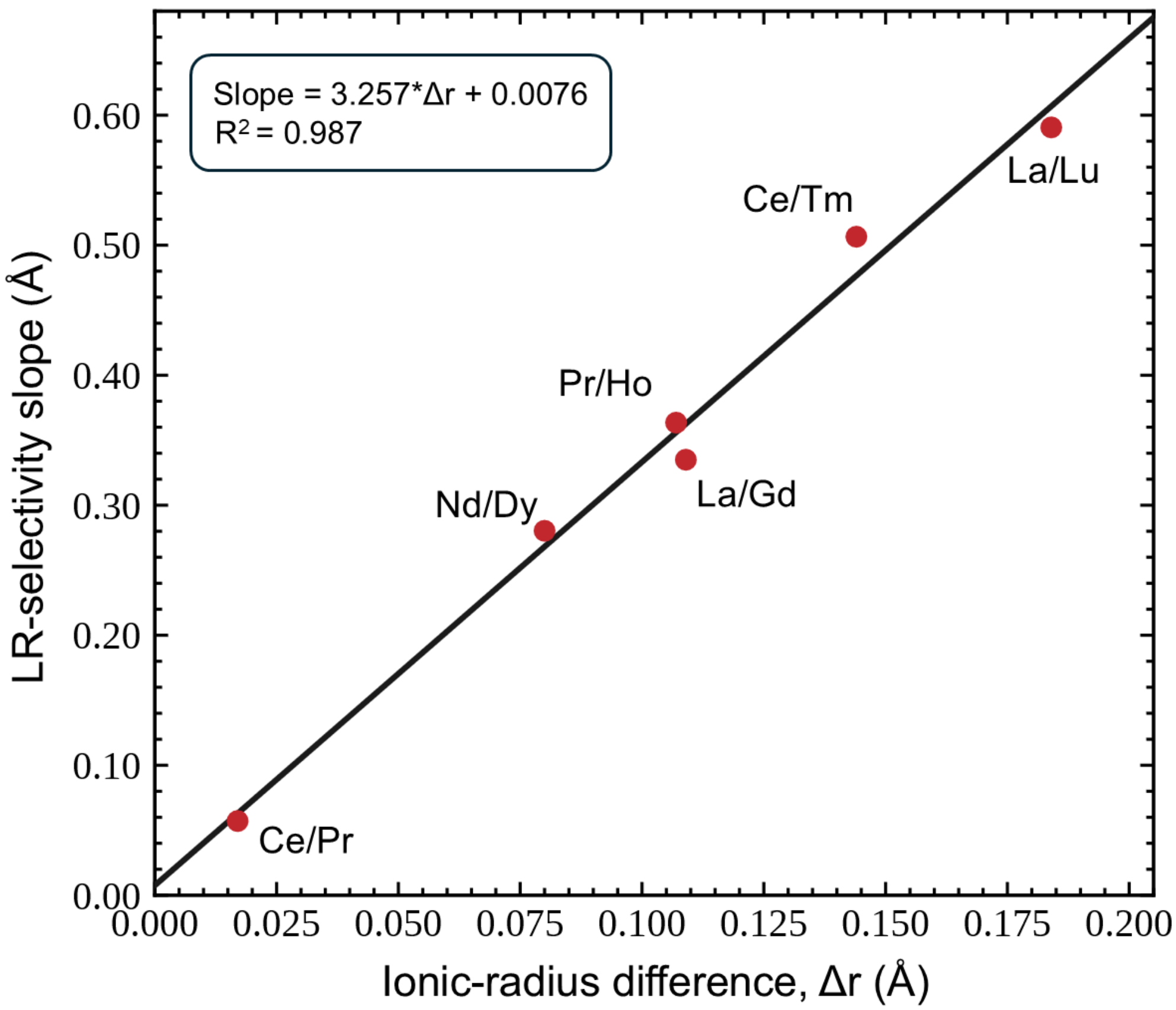

Figure S3. Dependence of the LR–selectivity slope on the ionic-radius difference, Δr.

As shown in Figure S3, the fitted slopes, $m_{A/B}$, of the $\Delta\Delta G$–LR relationships increase approximately linearly with the ionic-radius difference, Δr, across the six lanthanide pairs (R^2 = 0.987). The slope $m_{A/B}$ measures how strongly size selectivity responds to a given increase in ligand local rigidity for a particular lanthanide pair. Accordingly, pairs with larger ionic-radius mismatches show a stronger selectivity response to LR, whereas the adjacent Ce/Pr pair exhibits the shallowest dependence. The approximately linear scaling of $m_{A/B}$ with Δr supports, to first order, $m_{A/B} \approx k\Delta r$ (k = 3.257), and therefore an LR-dependent selectivity contribution proportional to $k\Delta r \cdot LR$.

S4. N···N Response during Cavity Compression

The O···O distance was used as the constrained coordinate in the cavity-compression scans, while all remaining geometric degrees of freedom were allowed to relax freely at each scan point. The N···N separation was monitored independently to determine how deformation of the O-donor cavity is transmitted to the N-donor framework.

To illustrate this response across structurally distinct ligand frameworks, five representative ligands were considered: DABPy, BLBPy, DAPhen, BLPhen, and BLPhen-B5. Across this subset, the N···N separation changes approximately linearly with O···O compression, but with a substantially smaller amplitude. The relationship was described by $d_{\mathrm{N\cdots N}} = \alpha d_{\mathrm{O\cdots O}} + \beta$, where α characterizes the magnitude of the N···N response to the imposed O···O deformation. The fitted α values span 0.0817–0.2407 (Table S3), corresponding to an N···N response of approximately 8–24% of the O···O displacement. The smallest responses are observed for the diamide ligands DAPhen and DABPy, with $\alpha = 0.0817$ and 0.0966, respectively, indicating that most of the imposed O···O compression can be accommodated through relaxation of the pendant amide groups with only a limited change in the N···N separation. Cyclization of these donors into the bis-lactam framework increases the coupling between the O- and N-donor coordinates, with α rising to 0.1864 for BLBPy and 0.2165 for BLPhen. Further annulation in BLPhen-B5 produces the largest response, $\alpha = 0.2407$, consistent with a more direct transmission of cavity compression through the fused donor-bearing framework.

Table S3. Linear-response parameters for the N···N separation during O···O cavity compression

Ligand	α	β, Å
**DABPy**	0.0966	2.1709
**BLBPy**	0.1864	1.7086
**DAPhen**	0.0817	2.2723
**BLPhen**	0.2165	1.5612
**BLPhen-B5**	0.2407	1.4347

The N···N distance remained unconstrained throughout all scans, and its relaxation is therefore already incorporated into the relaxed deformation-energy profile together with changes in donor orientation, backbone geometry, and other internal coordinates. The analysis shows that compression of the O-donor cavity is accompanied by a coupled relaxation of the N-donor framework without requiring N···N separation to be introduced as an additional constrained coordinate.

S5. Leave-One-Out Cross-Validation of the LR–Selectivity Relationship

The statistical robustness of the linear relationship between local rigidity (LR) and computed $\Delta\Delta G(\mathrm{La/Lu})$ was examined using leave-one-out cross-validation (LOOCV), influence diagnostics, and a targeted holdout test of the high-LR structures introduced in this work. For the complete thirteen-ligand dataset, linear regression gives $\Delta\Delta G(\mathrm{La/Lu}) = 0.5906\, LR + 0.1620$, with $R^2 = 0.9924$. The fitted slope is 0.5906 ± 0.0156Å (standard error), and the standard error

of the regression is 0.152 kcal/mol. The corresponding LR and $\Delta\Delta G$values are listed in Tables S1 and S2.

In LOOCV, each ligand was omitted in turn, the linear relationship was refitted using the remaining twelve ligands, and the $\Delta\Delta G(\mathrm{La/Lu})$value of the omitted ligand was predicted from its LR. This procedure gives $Q^2_{\mathrm{LOO}} = 0.9893$, $RMSE_{\mathrm{LOO}} = 0.166$ kcal/mol, and $MAE_{\mathrm{LOO}} = 0.141$ kcal/mol. For comparison, the in-sample RMSE of the full regression is 0.139 kcal/mol. Thus, omission of individual observations produces only a small increase in prediction error within the ligand space examined here.

Influence diagnostics were additionally used to determine whether the fitted relationship was controlled by individual ligands. Upon omission of each ligand in turn, the fitted slope varied only from 0.5815 to 0.6006 Å, corresponding to a maximum deviation of 1.7% from the full-dataset value. DAPhen-NH_2 gave the largest Cook's distance ($D = 0.360$), slightly exceeding the commonly used screening criterion $4/n = 0.308$. However, removal of DAPhen-NH_2 changes the fitted slope by only 1.5%, indicating limited practical influence on the regression. BLPhen-B5 is the highest-leverage point in the dataset ($h = 0.339$), as expected from its position at the high-LR end of the sampled range, yet its omission changes the slope by only 1.4%. These results show that neither the most influential observation nor the highest-leverage high-LR structure materially determines the fitted relationship.

Because the five BLPhen-Bn structures were introduced specifically to extend the sampled LR range, an additional targeted holdout test was performed. The relationship was fitted using only the eight ligands that were not introduced in the present work for this purpose, and the resulting model was then used without refitting to predict $\Delta\Delta G(\mathrm{La/Lu})$ for the five withheld BLPhen-Bn structures. The eight-ligand fit, $\Delta\Delta G(\mathrm{La/Lu}) = 0.5913\,LR + 0.1499,$ has $R^2 = 0.9842$. Its slope is essentially identical to that obtained from the complete dataset (0.5913 versus 0.5906 Å). The five withheld structures are predicted with an RMSE of 0.092 kcal/mol and an MAE of 0.090 kcal/mol. Notably, BLPhen-B7 (LR=10.45 kcal/mol/Å) and BLPhen-B5 (LR=11.62 kcal/mol/Å) lie beyond the upper LR limit of the eight-ligand fitting set (LR=10.14 kcal/mol/Å) and are nevertheless predicted with absolute errors of only 0.06 and 0.10 kcal/mol, respectively. Thus, the structures introduced to extend the high-rigidity regime broaden the sampled LR range but do not generate the observed LR–selectivity relationship.

These analyses establish the internal statistical robustness of the LR–selectivity relationship within the N,O-tetradentate ligand space examined here. They do not establish quantitative transferability of LR to unrelated donor sets or coordination motifs, which remains to be tested separately.

S6. Robustness of the LR–Selectivity Relationship across DFT Levels of Theory

To assess whether the LR–selectivity relationship depends on the electronic-structure method, the analysis was repeated for a representative seven-ligand subset using five additional DFT levels of theory: PBE-D4/def2-TZVP, B3LYP-D3BJ/def2-SVP, TPSS0-D3BJ/def2-SVP, TPSS-D3BJ/def2-SVP, and M06-2X/def2-TZVP. The subset comprised DAPhen, BLPhen, and five BLPhen-derived structures spanning the low-to-high rigidity region of the series (BLPhen-B2, BLPhen-B4, BLPhen-B6, BLPhen-B7, and BLPhen-B5). For each level of theory, both the cavity-deformation profile and the La/Lu differential binding free energy were recalculated

consistently, and the resulting LR–ΔΔG(La/Lu) relationship was analyzed independently. The original TPSS-D4/def2-TZVP results for the same seven-ligand subset were included as the reference. In all cases, the listed basis sets refer to the non-lanthanide atoms. The lanthanide treatment was kept unchanged, using the same Stuttgart–Dresden 4f-in-core pseudopotentials and corresponding valence basis sets for La and Lu. The SMD aqueous solvation model, quasi-RRHO treatment of Gibbs free energies, and cavity-compression and harmonic-fitting procedures were likewise retained as described in Sections S1.1–S1.5.

Although the absolute LR and ΔΔG values vary with the level of theory, the linear relationship between them is retained in every case. The fitted slopes range from 0.5342 to 0.6586 Å, while the coefficients of determination range from R^2=0.9882 to 0.9972. Leave-one-out cross-validation gives $Q^2_{\mathrm{LOO}} = 0.9708–0.9948$, and the maximum absolute regression residual ranges from 0.109 to 0.259 kcal/mol across the six computational protocols.

Table S4. Regression statistics for the LR–$\Delta\Delta G(\mathrm{La/Lu})$ relationship at different levels of theory.

Level of theory	Slope (Å)	Intercept (kcal/mol)	R^2	Q^2_{LOO}
PBE-D4/def2-TZVP	0.5342	1.0336	0.9972	0.9948
B3LYP-D3BJ/def2-SVP	0.5410	1.7820	0.9922	0.9842
TPSS0-D3BJ/def2-SVP	0.5577	0.7788	0.9885	0.9708
TPSS-D3BJ/def2-SVP	0.5589	0.6472	0.9882	0.9735
M06-2X/def2-TZVP	0.6586	0.7751	0.9955	0.9909
TPSS-D4/def2-TZVP (original)	0.5833	0.2198	0.9949	0.9898

Taken together, these results show that the central LR–selectivity relationship is robust to substantial changes in the electronic-structure treatment. The similar fitted slopes indicate that the magnitude of the selectivity response to increasing LR is largely preserved across the different levels of theory, while the consistently high R^2 values show that the linear form of the relationship remains intact. The high Q^2_{LOO} values further demonstrate that this behavior is not driven by individual ligands within the seven-member test set, and the small maximum residuals indicate that deviations from linearity remain minor for every computational protocol examined. Although the absolute LR and ΔΔG(La/Lu) values shift with the chosen functional, basis set, and dispersion treatment, these method-dependent changes do not alter the underlying structure–selectivity trend. The persistence of the same relationship across GGA, meta-GGA, hybrid-GGA, and hybrid-meta-GGA descriptions therefore indicates that the observed LR–selectivity correlation is not an artifact of the original TPSS-D4/def2-TZVP level of theory.

S7. Global Rigidity–Selectivity Relationship

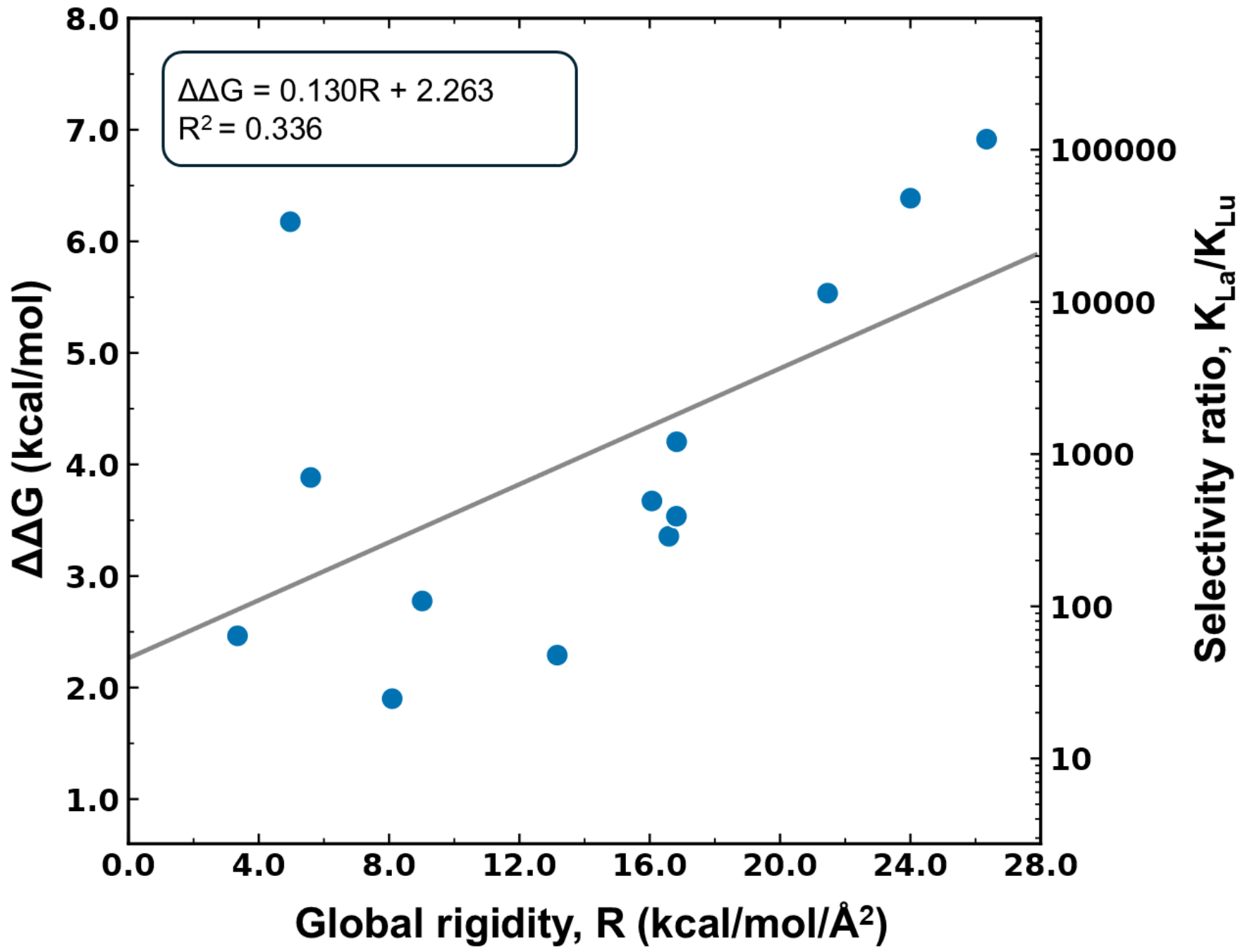

Figure S4. Global rigidity–selectivity relationship for the La/Lu pair. Relationship between computed $\Delta\Delta G(\mathrm{La/Lu})$ and global rigidity, R, across the thirteen ligands ($\Delta\Delta G = 0.130R + 2.263$, R^2=0.336).

To assess whether intrinsic framework stiffness alone captures the La/Lu selectivity trend, the computed ΔΔG(La/Lu) values were regressed against global rigidity, R. The resulting correlation was weak (R^2 = 0.336; Figure S4), in marked contrast to the strong relationship obtained with LR (R^2 = 0.992; Figure 3A). Thus, intrinsic cavity stiffness alone does not account for the observed selectivity trend; the displacement between the equilibrium and La-bound cavity dimensions must also be included, as incorporated in $LR = R(d^0 - d^*)$.

S8. Cartesian Coordinates

Cartesian coordinates for all optimized free ligands and $Ln(L)(NO3)_3$ complexes used in this work are provided in a single XYZ file accompanying the Supporting Information.

Supporting References

(1) Neese, F. The ORCA Program System. *WIREs Comput. Mol. Sci.* **2012**, *2* (1), 73–78. https://doi.org/10.1002/wcms.81.

(2) Neese, F. Software Update: The ORCA Program System—Version 6.0. *WIREs Comput. Mol. Sci.* **2025**, *15* (2), e70019. https://doi.org/10.1002/wcms.70019.

(3) Tao, J.; Perdew, J. P.; Staroverov, V. N.; Scuseria, G. E. Climbing the Density Functional Ladder: Nonempirical Meta–Generalized Gradient Approximation Designed for Molecules and Solids. *Phys. Rev. Lett.* **2003**, *91* (14), 146401. https://doi.org/10.1103/PhysRevLett.91.146401.

(4) Caldeweyher, E.; Ehlert, S.; Hansen, A.; Neugebauer, H.; Spicher, S.; Bannwarth, C.; Grimme, S. A Generally Applicable Atomic-Charge Dependent London Dispersion Correction. *J. Chem. Phys.* **2019**, *150* (15), 154122. https://doi.org/10.1063/1.5090222.

(5) Weigend, F.; Ahlrichs, R. Balanced Basis Sets of Split Valence, Triple Zeta Valence and Quadruple Zeta Valence Quality for H to Rn: Design and Assessment of Accuracy. *Phys. Chem. Chem. Phys.* **2005**, *7* (18), 3297–3305. https://doi.org/10.1039/b508541a.

(6) Weigend, F. Accurate Coulomb-Fitting Basis Sets for H to Rn. *Phys. Chem. Chem. Phys.* **2006**, *8* (9), 1057. https://doi.org/10.1039/b515623h.

(7) Dolg, M.; Stoll, H.; Savin, A.; Preuss, H. Energy-Adjusted Pseudopotentials for the Rare Earth Elements. *Theor. Chim. Acta* **1989**, *75* (3), 173–194. https://doi.org/10.1007/BF00528565.

(8) Dolg, M.; Stoll, H.; Preuss, H. A Combination of Quasirelativistic Pseudopotential and Ligand Field Calculations for Lanthanoid Compounds. *Theor. Chim. Acta* **1993**, *85* (6), 441–450. https://doi.org/10.1007/BF01112983.

(9) Yang, J.; Dolg, M. Valence Basis Sets for Lanthanide 4f-in-Core Pseudopotentials Adapted for Crystal Orbital Ab Initio Calculations. *Theor. Chem. Acc.* **2005**, *113* (4), 212–224. https://doi.org/10.1007/s00214-005-0629-0.

(10) Stoychev, G. L.; Auer, A. A.; Neese, F. Automatic Generation of Auxiliary Basis Sets. *J. Chem. Theory Comput.* **2017**, *13* (2), 554–562. https://doi.org/10.1021/acs.jctc.6b01041.

(11) Marenich, A. V.; Cramer, C. J.; Truhlar, D. G. Universal Solvation Model Based on Solute Electron Density and on a Continuum Model of the Solvent Defined by the Bulk Dielectric Constant and Atomic Surface Tensions. *J. Phys. Chem. B* **2009**, *113* (18), 6378–6396. https://doi.org/10.1021/jp810292n.

(12) Grimme, S. Supramolecular Binding Thermodynamics by Dispersion-Corrected Density Functional Theory. *Chem. – Eur. J.* **2012**, *18* (32), 9955–9964. https://doi.org/10.1002/chem.201200497.

(13) Pracht, P.; Grimme, S.; Bannwarth, C.; Bohle, F.; Ehlert, S.; Feldmann, G.; Gorges, J.; Müller, M.; Neudecker, T.; Plett, C.; Spicher, S.; Steinbach, P.; Wesołowski, P. A.; Zeller, F. CREST—A Program for the Exploration of Low-Energy Molecular Chemical Space. *J. Chem. Phys.* **2024**, *160* (11), 114110. https://doi.org/10.1063/5.0197592.

(14) Pracht, P.; Bohle, F.; Grimme, S. Automated Exploration of the Low-Energy Chemical Space with Fast Quantum Chemical Methods. *Phys. Chem. Chem. Phys.* **2020**, *22* (14), 7169–7192. https://doi.org/10.1039/C9CP06869D.

(15) Bannwarth, C.; Ehlert, S.; Grimme, S. GFN2-xTB—An Accurate and Broadly Parametrized Self-Consistent Tight-Binding Quantum Chemical Method with Multipole Electrostatics and Density-Dependent Dispersion Contributions. *J. Chem. Theory Comput.* **2019**, *15* (3), 1652–1671. https://doi.org/10.1021/acs.jctc.8b01176.

(16) Ehlert, S.; Stahn, M.; Spicher, S.; Grimme, S. Robust and Efficient Implicit Solvation Model for Fast Semiempirical Methods. *J. Chem. Theory Comput.* **2021**, *17* (7), 4250–4261. https://doi.org/10.1021/acs.jctc.1c00471.
(17) Summers, T. J.; Taylor, M. G.; Augustine, L. J.; Janssen, J.; Perez, D.; Batista, E. R.; Yang, P. On the Importance of Configuration Search to the Predictivity of Lanthanide Selectivity. *JACS Au* **2025**, *5* (2), 631–641. https://doi.org/10.1021/jacsau.4c00770.
(18) Ustynyuk, Y. A.; Zhokhova, N. I.; Gloriozov, I. P.; Matveev, P. I.; Evsiunina, M. V.; Lemport, P. S.; Pozdeev, A. S.; Petrov, V. G.; Yatsenko, A. V.; Tafeenko, V. A.; Nenajdenko, V. G. Competing Routes in the Extraction of Lanthanide Nitrates by 1,10-Phenanthroline-2,9-Diamides: An Impact of Structure of Complexes on the Extraction. *Int. J. Mol. Sci.* **2022**, *23* (24), 15538. https://doi.org/10.3390/ijms232415538.
(19) Evsiunina, M. V.; Khult, E. K.; Matveev, P. I.; Kalle, P.; Lemport, P. S.; Petrov, V. S.; Aksenova, S. A.; Nelyubina, Y. V.; Koshelev, D. S.; Utochnikova, V. V.; Petrov, V. G.; Ustynyuk, Y. A.; Nenajdenko, V. G. Unravelling the Mechanism of F-Element Extraction by Phenanthroline-Diamides: A Case of 4,7-Substituted 1,10-Phenanthroline-2,9-Diamides. *Sep. Purif. Technol.* **2024**, *339*, 126621. https://doi.org/10.1016/j.seppur.2024.126621.
(20) Ivanov, A. S.; Bryantsev, V. S. A Computational Approach to Predicting Ligand Selectivity for the Size-Based Separation of Trivalent Lanthanides. *Eur. J. Inorg. Chem.* **2016**, *2016* (21), 3474–3479. https://doi.org/10.1002/ejic.201600319.
(21) Pozdeev, A. S.; Ivanov, A. S.; Jansone-Popova, S.; Jiang, D. Tuning Lanthanide Binding with Phenanthroline-Based Diamides via Electron-Donating and Electron-Withdrawing Groups. *J. Phys. Chem. A* **2025**, *129* (43), 9863–9870. https://doi.org/10.1021/acs.jpca.5c03506.
(22) Shannon, R. D. Revised Effective Ionic Radii and Systematic Studies of Interatomic Distances in Halides and Chalcogenides. *Acta Cryst* **1976**, *32*, 751–767.
